# A User Manual to Measure Gas Diffusion Kinetics in Plants: Pneumatron Construction, Operation, and Data Analysis

**DOI:** 10.3389/fpls.2021.633595

**Published:** 2021-06-07

**Authors:** Christophe L. Trabi, Luciano Pereira, Xinyi Guan, Marcela T. Miranda, Paulo R. L. Bittencourt, Rafael S. Oliveira, Rafael V. Ribeiro, Steven Jansen

**Affiliations:** ^1^Institute of Systematic Botany and Ecology, Ulm University, Ulm, Germany; ^2^Center R&D in Ecophysiology and Biophysics, Agronomic Institute (IAC), Campinas, Brazil; ^3^Department of Plant Biology, Institute of Biology, University of Campinas (UNICAMP), Campinas, Brazil; ^4^College of Life and Environmental Sciences, University of Exeter, Exeter, United Kingdom

**Keywords:** vulnerability to embolism, plant hydraulics, plant pneumatics, plant water relations, gas flow, gas diffusion, xylem

## Abstract

The Pneumatron device measures gas diffusion kinetics in the xylem of plants. The device provides an easy, low-cost, and powerful tool for research on plant water relations and gas exchange. Here, we describe in detail how to construct and operate this device to estimate embolism resistance of angiosperm xylem, and how to analyse pneumatic data. Simple and more elaborated ways of constructing a Pneumatron are shown, either using wires, a breadboard, or a printed circuit board. The instrument is based on an open-source hardware and software system, which allows users to operate it in an automated or semi-automated way. A step-by-step manual and a troubleshooting section are provided. An excel spreadsheet and an R-script are also presented for fast and easy data analysis. This manual aims at helping users to avoid common mistakes, such as unstable measurements of the minimum and maximum amount of gas discharged from xylem tissue, which has major consequences for estimating embolism resistance. Major advantages of the Pneumatron device include its automated and accurate measurements of gas diffusion rates, including highly precise measurements of the gas volume in intact, embolised conduits. It is currently unclear if the method can also be applied to woody monocots, gymnosperm species that possess torus-margo pit membranes, or to herbaceous species.

## Introduction

The Pneumatron is a device that allows automated measurements of the gas diffusion kinetics in plant xylem tissue (Pereira et al., 2020a). Pneumatic measurements have been applied to xylem tissue of various plant organs, such as stems (Pereira et al., 2016; Zhang et al., 2018; Jansen et al., 2020; Paligi et al., 2021), roots (Wu et al., 2020), and leaves (Pereira et al., 2020a; Guan et al., 2021), to estimate vulnerability to hydraulic failure of the water conducting cells, which is especially relevant to plants that undergo severe drought stress. Although the device has been designed to address questions in the field of xylem anatomy and physiology, it is expected that the instrument can also be applied to a wide range of other porous media.

During a pneumatic measurement, a partial vacuum is pulled in a discharge tube to extract gas from xylem tissue of a cut branch, petiole, or root during less than 1 min (Pereira et al., 2016; Bittencourt et al., 2018). The amount of gas extracted can be calculated from pressure measurements, using the ideal gas law. As gas extraction from embolised, intact vessels is fast and delays in measuring pressure changes can increase the measuring error (Paligi et al., 2021; Yang et al., 2021), the Pneumatron has been shown to provide a major improvement of the manual pneumatic apparatus. The main advantages of the Pneumatron are its automated approach and high speed in milliseconds of creating a partial vacuum, taking pressure measurements, storing data, and opening or closing the valves between the discharge tube and the atmosphere. By applying repetitive measurements over time, and combining these pneumatic data with a quantification of sample dehydration, a “vulnerability curve” can be obtained in a straightforward way without any time-consuming analyses. For a general understanding of the manual pneumatic method, we refer to earlier papers (Pereira et al., 2016, 2020a; Bittencourt et al., 2018; Zhang et al., 2018; Jansen et al., 2020).

So far, pneumatic vulnerability curves have been conducted for hundreds of species by a few research groups (Pereira et al., 2016, 2020a, 2021; Zhang et al., 2018; Barros et al., 2019; Brum et al., 2019; Oliveira et al., 2019; Bittencourt et al., 2020; Giles et al., 2020; Sergent et al., 2020; Chen et al., 2021; Guan et al., 2021; Paligi et al., 2021). Although its construction is simple and based on an accessible and open-source platform (Arduino), there is a need for a detailed manual with clear instructions on the construction of a Pneumatron device, its operation, and the analysis of pneumatic data. Such details are crucial to introduce new research groups to pneumatic measurements, which are considerably different from measurements of hydraulic conductivity, and to ensure accurate and correct interpretation of the data obtained. This paper aims to provide such a user manual, which may avoid common mistakes in pneumatic experiments and misinterpretation of data. Besides its importance for measuring xylem embolism resistance, the Pneumatron can also be used to study gas kinetics of plants *in vivo*, and vessel length distributions (Pereira et al., 2020b; Yang et al., 2021). These methods require minor modifications of the software programme and tube connections. Contrary to other standard methods on embolism resistance, the Pneumatron device is very fast and user-friendly, which makes this device also useful for field measurements at remote places.

## Materials and Equipment

### Equipment

**FIGURE 1:**
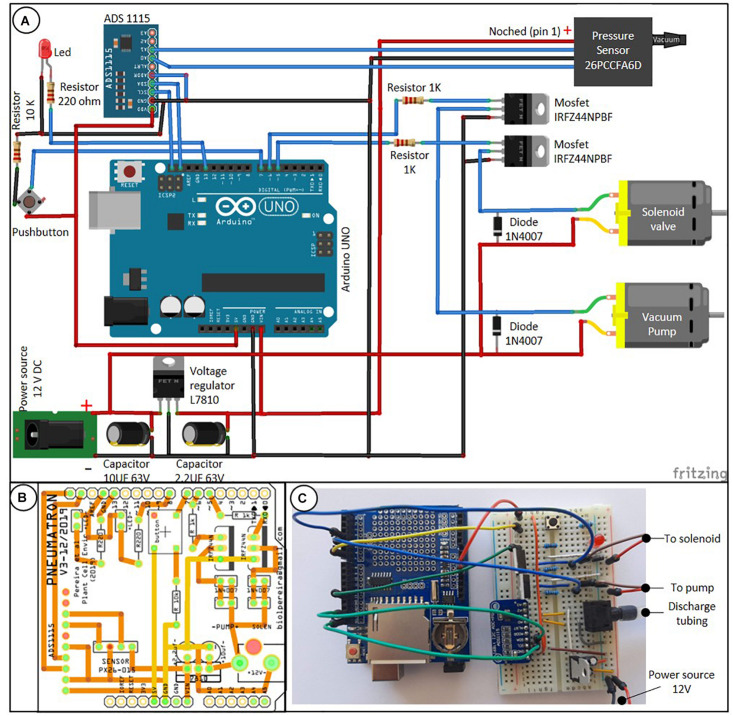
Connection scheme between the Pneumatron components based on wires [**(A)** the data logger module, which is connected on top of the Arduino board, is not shown], a two-layer printed circuit board (PCB) of a wireless Pneumatron shield (https://github.com/Pneumatron/construction) **(B)**, and a non-permanent assembly using a breadboard **(C)**. The complete list of all components is listed in Supplementary Table 1 and a schematic connection among the parts is presented in Supplementary Figure 1.

The Pneumatron is composed of a microcontroller system (Atmega328P, Microchip, on an Arduino^®^ Uno board), a data storage (SD card) and real time clock (DS1307, Maxim Integrated; both assembled on an Adafruit^®^ Data Logger Shield), a 16 bits analog-to-digital converter (ADS1115, Texas Instrument), a vacuum pump, a solenoid valve (3/2 connection, normally closed) and its driver (a logic-level N-channel mosfet plus flyback diode) (Figure 1A). These components are easily available in electronic shops.

A printed circuit board (PCB) to construct the Pneumatron Shield (Figure 1B) may be used to facilitate accurate connections between all electronic parts, to reduce connection problems, and to provide appropriate isolation and high resistance to damage, while requiring also minimal soldering skills. The PCB can be easily manufactured via several PCB producers by using the production files (Gerber Files; available in https://github.com/Pneumatron/construction). Alternatively, it is possible to use a breadboard to make all connections (Figure 1C), although this is not recommended, as it increases the chance of failure due to bad connections and, if not properly done, can lead to voltage gradients in the analog-to-digital converter and sensor supply, and to grounding. A breadboard is not a permanent option, but can be useful for testing or for a low-demand experiment.

The individual components of the Pneumatron are listed in Supplementary Table 1. The assembly and testing do not require specialised knowledge, but correct orientation of some components must be respected (Figure 1A). For protecting the sensitive parts of the Pneumatron, it is advised to enclose these into a box. It is recommended to do this for preventing any damage to the vacuum pump, solenoid valve, and the potential short circuit.

**FIGURE 2:**
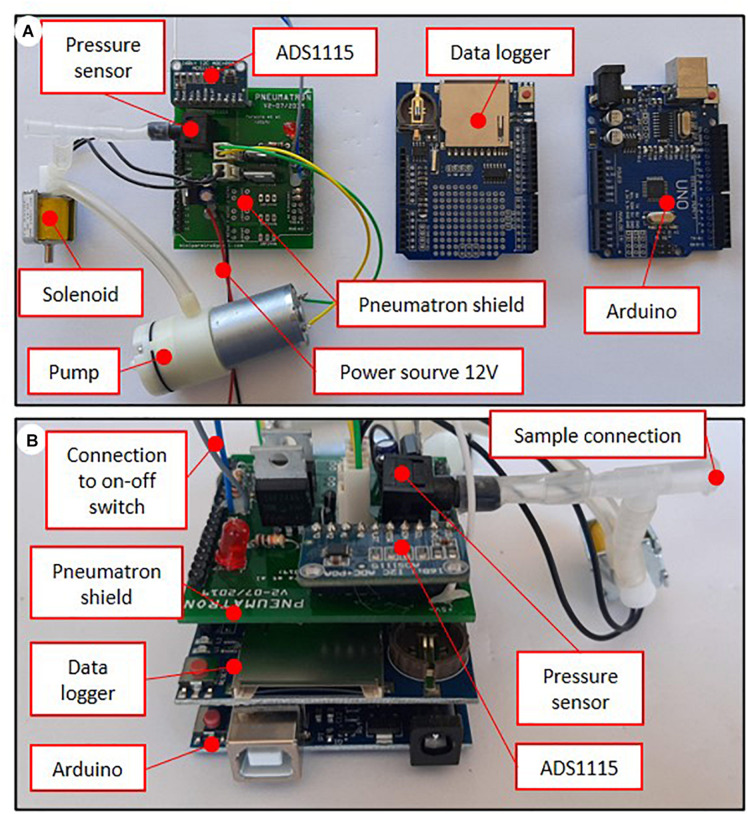
Components and connections **(A)**, and assembling position **(B)** of the Pneumatron with a Pneumatron shield (in which the switch and power source were connected using wires).

After assembling the electronic parts (Figure 1), the pressure sensor, the vacuum pump, and the solenoid valve need to be connected to each other with silicone tubing (Figure 2A). These components are then connected to the PCB circuit board (Figure 2A). After assembling the pins onto the data logger shield and installing the different components onto the PCB board, it is necessary to assemble them into a stacked-up structure with the Arduino at the bottom, the data logger shield in the middle, and the Pneumatron shield at the top (Figure 2B).

### Software

The Pneumatron is built on an Arduino^®^ Uno or similar board. To be able to upload or modify the programmes, installation of software is required. Further information can be found on the Arduino web-site^1^. System information and requirements are needed to get the correct version of the programme, depending on which platform the software is to be installed on.

In order to get the programme fully functioning, some of the libraries need to be installed or updated. The libraries used in this version of the Pneumatron are the SPI and Wire (v. 2.3.5) for SPI and I2C communication, RTCLib (version 1.2.4) for using the DS1307 real-time clock, Adafruit_ADS1X15 (v. 1.0.1) for using the ADS1115 analog-to-digital converter, and the SD library (v. 2.3.5) for using the SD card with a FAT32 file system. More information can be found at: https://www.arduino.cc/en/guide/libraries. Arduino novices might require further training on how to upload a given programme onto the board. Some complementary information is available at: https://www.arduino.cc/en/main/howto.

The programmes for the Pneumatron can be downloaded from https://github.com/Pneumatron/software. After uploading the programmes, the equipment is ready for calibration and testing.

### Programmes

#### Automated Mode

**FIGURE 3:**
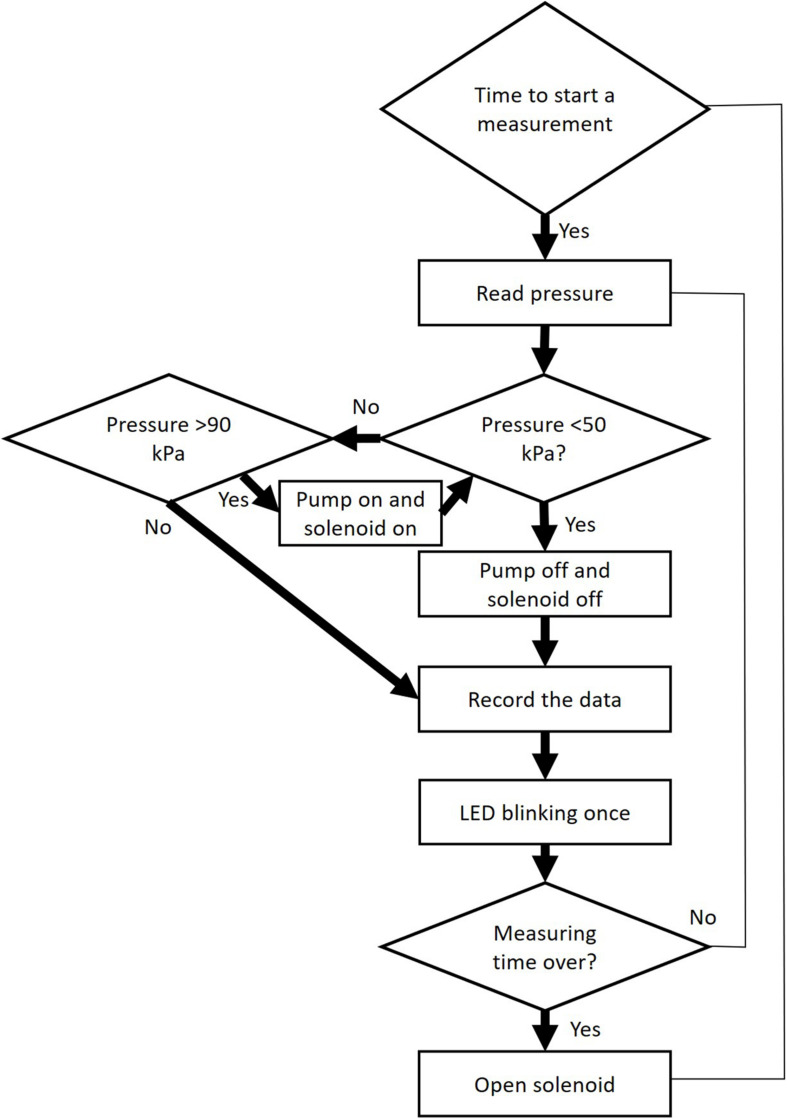
Workflow of operational steps when using the Pneumatron in automated mode. This workflow is repeated many times within a pre-selected measuring interval (≥15 s).

The programme controls the pump and the solenoid valve, and reads data from the pressure sensor via an ADC 16 bits converter (Figure 3). Firstly, the pump will be heard creating a partial vacuum inside the discharge tubing (ca. 40 kPa of absolute pressure), and should achieve this partial vacuum in less than 1 s. If not, either the vacuum pump is not working well, or there is a leakage. After reaching the target pressure, it will stop and pressure values will be recorded for 1 min, which is the default measuring time. However, it should be emphasized that the best results for constructing vulnerability curves are obtained for an extraction period of 15 s based on modelling (Yang et al., 2021) and experimental work (Paligi et al., 2021). Users should change the duration of the gas extraction, depending on the experimental requirements, and should consider that long extraction periods (>30 s) increase the proportion of gas from other sources than embolised, intact conduits, such as gas dissolved in xylem sap, or gas extracted radially across cell walls. During this measuring period, the LED will flash every 0.5 s. Finally, the pressure inside the apparatus will return to atmospheric pressure via repeated opening and closing of the solenoid valve over a short time period (5 s), and the device will then wait for the next measurement to be taken (e.g., every 15 min). The suggested minimum time interval between measurements is 15 min, which allows the gas in the xylem tissue to reequilibrate with atmospheric pressure before the next measurement is taken.

#### Semi-Automated Mode

The semi-automated mode is similar to the automated one, but each individual measurement has to be activated manually by the operator using a switch. The rest of the semi-automated process is similar to the automated method, except for activation of the solenoid valve at the end of a measurement. The switch to take a measurement can only be pressed after an entire measuring cycle has been finished.

## Methods

### Testing and Calibration

#### Testing the Pneumatron for Leakage

After assembling the Pneumatron, the device should be tested for leakage. This is achieved by blocking off the tubing that is used for the connection to a sample, while running the programme for several measurement cycles in the semi-automated mode. If the pump is working permanently, or frequently (e.g., more than once during a single measurement), we refer to the troubleshooting section (Supplementary Material 1). Then, the device is powered off and the SD card is removed. An ideal and fast way to test the data obtained is to import the csv file into Microsoft Office Excel^®^ and to select only the data column on the right, plotting the gas extraction data into a scatter point graph. The data should be as close to constant as possible (within 1–2%), apart from the first point, which is close to zero. If the data seem to be drifting more than 5% and are decreasing regularly with each measurement, there is a leakage. More information about how to deal with leakages can be found in the troubleshooting section.

#### Calibration

**FIGURE 4:**
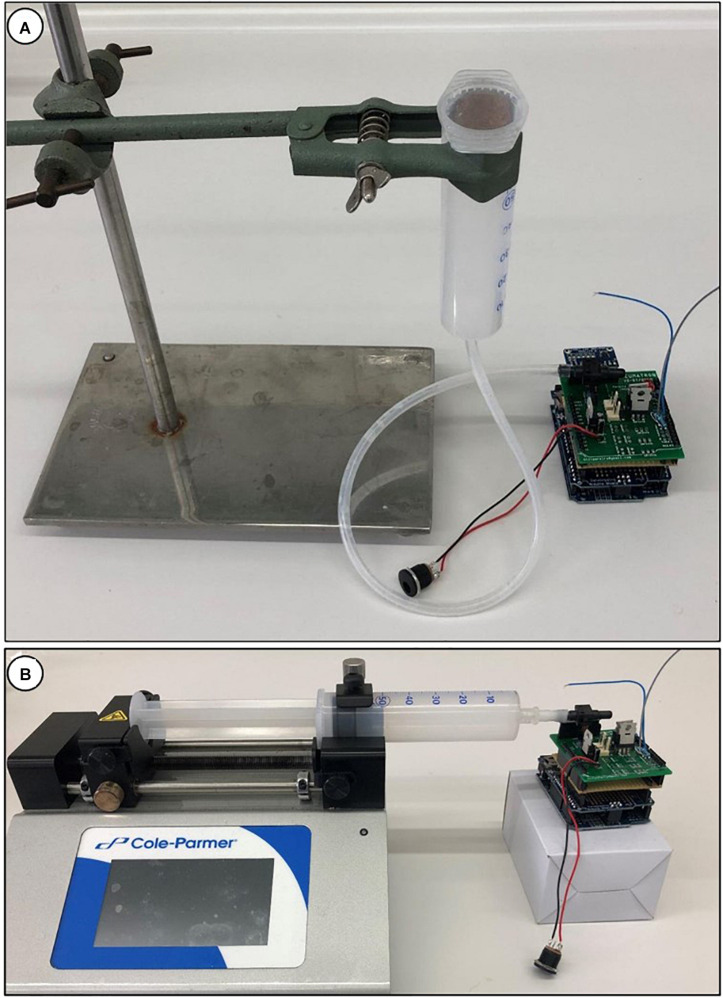
Calibration of the pressure sensor in a Pneumatron device using a water column **(A)**, with a water-filled syringe and tubing; the pressure is estimated by measuring the height of the water column (from the pressure sensor to the free surface of the liquid). Calibration of a pressure sensor can also be done using a syringe pump **(B)**.

The Pneumatron measurements are based on relative values (percentage of air discharge) calculated from the difference between the initial and the final pressure measured in the discharge tubing. For this reason, the unit used (volts or kPa) is not critical. However, if a correct pressure unit is required for a different purpose, it is necessary to calibrate the pressure sensor instead of using the equation of the manufacturer (default in the Pneumatron programmes) since the voltage/pressure ratio changes with the voltage supplied. To calibrate the sensor, use the calibration software^2^. There are two easy methods for calibration, one relying on the height of a water column (Figure 4A), and the other one using a syringe pump (Figure 4B).

##### Water column calibration

The pressure sensor should first be disconnected from the valve and pumping system, and is then connected to a tube with a syringe (see Figure 4A). The pressure sensor, the tubing, and the syringe should be fully filled with water. Make sure that there is no air bubble left in the pressure sensor. This can be done by using a needle, but make sure the membrane of the sensor is not punctured. The water-filled syringe is then fixed at a certain height. The distance between the pressure sensor and the water surface of the liquid in the syringe can then be measured, and the pressure is recorded. This process is repeated at two or three height levels.

When the measurements have been taken, the file can be copied from the Pneumatron and the csv data can be checked, for instance using Microsoft Office Excel^®^. The pressure measurements should be represented by a step graph and the value for each step should be linked with the height of the water column. To determine the pressure that is produced by the water column, the following equation is used:

(1) P=ρgh

Where ρ is the density of the liquid used (1,000 kg m^–3^ for water), *g* is the acceleration of gravity (9.81 m s^–2^) and *h* is the height (m) between the pressure sensor and the free surface of the liquid in the syringe. The resulting pressure will be in Pa and can be converted to kPa for ease of use (1,000 Pa = 1 kPa).

The calibration equation is obtained by fitting the applied pressure (Y-axis) to the sensor voltage measured (X-axis). This equation (the slope and intercept values) should then be included in the programme (see instructions at the initial lines of the programme), and the programme is uploaded once more onto the Arduino board.

##### Syringe pump calibration

The Pneumatron is switched on, and the air contained in the 60 mL syringe is compressed. The pressure measured is relative. When air in the 60 mL syringe is compressed to 50 mL, the pressure is then 20 kPa; at 40 mL, the pressure is 50 kPa, and 100 kPa is reached at 30 mL. The same process as the approach with the water column is followed to determine the calibration equation and to upload the equation to the Arduino board.

##### Implementation of the calibration parameters

Depending on the manufacturer of the pressure sensor, the sensor response for a given pressure will be different. In case the pressure sensor differs from the one suggested here, the datasheet should provide information about the response of the sensor to the pressure applied, as well as about the power supplied to the sensor, which is 10 volts in the Pneumatron. Please refer to the datasheet of the sensor and check the equation to be implemented in the programme (see footnote text 3), as shown in section “Water Column Calibration”.

### Experimental Techniques

#### Sample Preparation

Unlike sampling for hydraulic measurements, plant material can be cut in air because the conduits that are cut open need to be filled with air on purpose. The cut open conduits therefore represent an extension of the discharge tube (Pereira et al., 2016; Jansen et al., 2020). The length of samples should preferentially be longer than the maximum vessel length (Greenidge, 1952), which is unproblematic if terminal organs such as terminal branches, roots, or single leaves are used as they are part of the ending or starting point of the xylem transport system, and they are not showing another cut. However, short samples, for instance stem segments with one or two end walls between vessels, should be avoided. Besides having at least one cut end, both terminal samples and segments should ideally be intact, and any damage due to natural or artificial wounds should be avoided as this could lead to air entry and embolism spreading under relatively high xylem water potentials (Guan et al., 2021; Paligi et al., 2021). It is possible to work with stem or root segments, as long as air-entry is prevented at one of the cut sides (Wu et al., 2020). The distal end of a segment could, for instance, be blocked with super glue or a resin to avoid gas exchange. Stem or root segments, however, take much longer to dehydrate than a terminal branch with leaves.

After cutting in air, samples should be covered up with a black plastic bag to avoid dehydration. Since it is important to start pneumatic measurements on samples that are well hydrated, samples need to be collected early in the morning, preferentially during the wet season. However, if these conditions are not possible, samples can be left within the plastic bag with their cut ends in water for several hours or one night before taking measurements.

#### Determination of the Discharge Tubing Volume

A crucial factor that determines the precision of the Pneumatron is the volume of the discharge tubing, which needs to be proportional to the amount of gas discharged from the xylem (Pereira et al., 2020a). To estimate the volume of the discharge tube, completely dehydrated samples can be used, of similar size to samples of interest. This approach will allow users to measure the maximum amount of gas that can be discharged. Adjusting the discharge tube volume is essential and could prevent problems caused by two different situations. Firstly, if this volume is too small, the pressure will quickly reach atmospheric pressure, especially when the sample becomes considerably dehydrated. The current Pneumatron programme pulls a new partial vacuum when a pressure of 90 kPa has been reached, and interrupts the measurements that need to be taken within a certain time interval (at least 15 s, or slightly longer). Secondly, if the discharge volume is too large, the measuring error of the pressure sensor will be relatively large (see Figure 4 in Jansen et al., 2020; Pereira et al., 2020a). Repetitive measurements over 15 min time intervals are required to make sure that the maximum amount of gas represents a constant plateau. Based on empirical evidence, the ideal volume of the discharge tube can be estimated by dividing the maximum volume of gas discharged in microliters by 510.2 (Pereira et al., 2020a), which gives the volume in milliliters that maximizes the Pneumatron precision.

Alternatively, it is possible to take a few measurements on a fully dehydrated plant organ using a small tubing volume (<1 mL) and to check if the vacuum pump will restart during the measurement period. The vacuum pump will automatically be activated again once a pressure of 90 kPa has been reached, although users can change this threshold. If the vacuum pump restart, the tubing volume should be increased and tested again. This step needs to be repeated until the pump is no longer reactivated within the desired measuring interval (≥15 s). The volume can be adjusted by adding or removing a certain amount of rigid tubing. It is also important that the tubing has a constant volume under the partial vacuum that is pulled. See Supplementary Table 1 for tubing details.

#### Always Test for Leakage Before Starting With Measurements

Close off the open tube of the Pneumatron, and insert an empty SD card to the data logger shield. Connect the Pneumatron to a power supply system. If the automated mode of the programme is used, the vacuum pump will immediately be heard. For the semi-automated mode, the measurements will start only when the button is pushed. Then, wait until the first measurement cycle (1 min by default) has finished. The LED light flashes when pressure data are being recorded, and stops when the measurement has finished. Then, remove the SD card, and check the data to see if there was any potential leakage during the measurement (see details in how to get and analyse data, and the troubleshooting part for potential leakage problems).

#### Cleaning the Memory Card

Because measuring data are always stored in the file “log.txt”, it is important to clean the memory card before new measurements are recorded. Make sure also that the SD card has been inserted properly (correct orientation and tight insertion).

#### Connecting a Sample to the Pneumatron

It is recommended to start with hydrated plant material, preferentially bagged to avoid rapid dehydration at the beginning. Trim the cut end of the sample carefully with a fresh razor blade to have all conduits nicely cut open. Not carefully cutting open the embolised conduits at the sample end may cause resistance to gas extraction. Cutting should be done in air as cut-open conduits should intentionally be gas-filled (i.e., embolised). When working with non-terminal branches, leaves, or roots, make sure that the length of the sample is longer than the maximum vessel length. Otherwise, a high amount of air will be sucked up via the cut-open conduit. As mentioned above, it might be important to apply glue to distal cuts or broken twigs to avoid gas exchange via cut or damaged xylem. Altought it is possible that vessels at these cut or broken xylem tissues are not directly connected to the cut-open xylem tissue to which the Pneumatron is connected, it is recommended to apply glue to these places (Guan et al., 2021; Paligi et al., 2021).

Connect the branch end to the Pneumatron with elastic tubing (see Supplementary Table 1 for details of the tubing and clamps), and choose the best-fitting tube, considering the size of the cut sample end. Use parafilm, plastic clamps, or glue to ensure that there is no leakage.

Since dehydration of fresh samples is affected by environmental conditions, pneumatic measurements should ideally be performed under conditions of stable temperature and humidity. As initial dehydration is typically fast, samples can be bagged (or semi-bagged). This is especially recommended when working with species that are rather vulnerable to embolism, and will allow the user to collect more data points with the Pneumatron, especially at water potentials that are not very negative.

#### Turning on the Apparatus

Connect the apparatus to the power supply and the measurements will start automatically when the automatic mode is running. Measurements will be taken at every 15 min by default, but the time interval can easily be changed in the programme. Calculating the volume of air discharged for each measurement is discussed below in the “Data Analysis” section.

#### Stopping the Measurements

Measurements can be stopped when the branches are completely dehydrated (see the next section about water potential measurements). After severe dehydration, the maximum amount of gas extracted from the plant sample has been achieved, and there will be no longer increases in the percentage of gas discharged with further dehydration. Then, a curve between air discharge and time or, preferentially water potential, will form a plateau.

**FIGURE 5:**
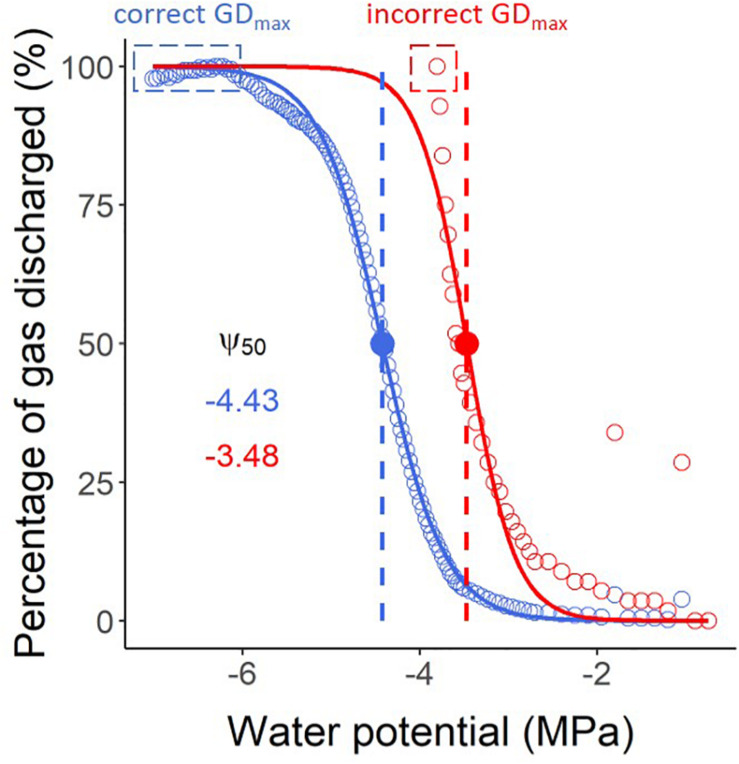
Vulnerability curves estimated with a Pneumatron for *Olea europea*, considering both stable maximum gas discharge (GD_max_) measurements (a plateau below -6 MPa, in blue) and an incorrect value as GD_max_ (in red), without a plateau. The water potential corresponding to 50% of the maximum amount of gas extracted (Ψ_50_, in MPa) is shown with the same colors. The red curve and its Ψ_50_ value should not be interpreted as accurate measurements due to finishing the pneumatic experiment too early, and therefore estimating GD_max_ incorrectly.

It is a common mistake to stop the pneumatic measurements too early before a stable plateau of maximum gas extraction has been achieved. Unlike the bench dehydration method and the flow-centrifuge method, which allow users to identify the percentage of loss of hydraulic conductivity during measurements, it is not possible to determine the percentage of gas discharged (PGD, %) during the experiment. PGD values are normalised by the minimum and maximum amounts of gas extracted, and therefore can only be calculated when the maximum volume of extracted gas has been identified. When users stop their pneumatic measurements too early, the Ψ_50_ (water potential in which 50% of xylem embolism is formed) can be strongly underestimated as simulated in Figure 5. When working with a sample that has leaves, it is recommended to run the measurements until the leaves become crispy. It is also important to avoid leakages caused by stem shrinkage, tightening the clamp, and reapplying glue at the connection with the Pneumatron.

#### Taking Water Potential Measurements

When plotting vulnerability curves, xylem water potentials are required for the X-axis. Xylem water potential can be measured in different ways, with a pressure chamber or psychrometer.

##### Measuring water potential with a pressure chamber

The branches need to be bagged at least 30 min before measurements are taken. If the whole branch is put in a dark environment (black plastic bag), the xylem water potential of a branch should be in equilibrium with the leaf water potential. Then, leaf water potential can be assumed to equal the xylem water potential.

Excise one or two leaves from a branch and measure the leaf water potential using a pressure chamber. After cutting the leaves from the branch, it is important to apply glue to seal the cuts, because even cut petioles may contain vessels that run directly into the stem xylem and could lead to artificial air entry (Choat et al., 2016). Then, the branches are left to dehydrate and new measurements are taken. Write down the water potential results and the exact time of the measurement, so that water potential data and pneumatic measurements can be matched later.

Although the Pneumatron generally takes measurements at 15 min time intervals, it is practically not feasible to take water potential measurements with a pressure chamber at such high frequency. Therefore, estimating the decline in xylem water potential over time by interpolation is recommended (Pereira et al., 2020a). Generally, water potential measurements should be taken more frequently during the start of the dehydration process, and longer time periods can be taken once stomata have closed and a linear decline in water potential has been obtained. Thus, usually five to ten measurements are enough to estimate the water potential decline over the entire dehydration period, depending also on the number of leaves that are available on the branches studied.

##### Measuring water potential with a stem/leaf psychrometer

Leaf or stem psychrometers can be used, also in combination with pressure chamber measurements. The great advantage of using psychrometers, is that once they have been installed, the water potential is automatically monitored at a certain time interval, which could be set to the same time interval as the Pneumatron measurements.

## Results

### Data Extraction and Analysis of the Vulnerability Curves Using an R Script

An R script^3^ can be used to analyse the data. For this, the raw data of the Pneumatron (log.csv file) and another file with water potential data are needed, saved as a comma delimited file (csv). Headers of three columns must be defined: “date”, “hour”, and “psy” [date and hour, in the format of dd/mm/yyyy and hh:mm, respectively, and water potential measurements (psy)]. Then, important input are the experimental conditions as indicated at the beginning of the R script (file name and address, time to reach the initial and final pressure measurements, reservoir volume, and atmospheric pressure). After running the script in R, the water potential between two consecutive measurements will be estimated, assuming a linear decrease over time. For example, based on six measurements over 2 days of branch dehydration (with three measurements at 2 h intervals during early dehydration, and three measurements every 5–8 h during later stages), the water potential will be estimated at an interval of 15 min. In this way, the pneumatic measurements and estimated xylem water potentials are obtained at the same temporal scale.

**FIGURE 6:**
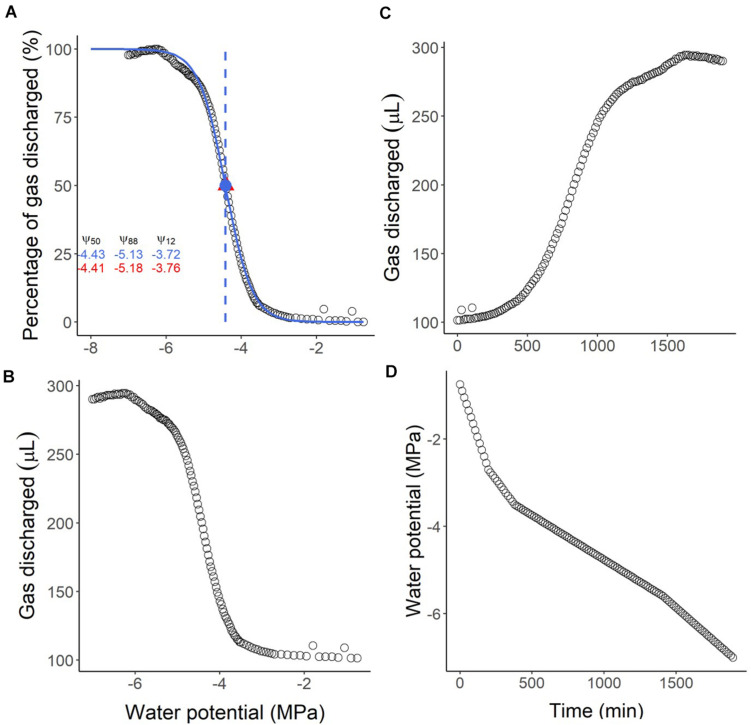
Example of output graphs from the R-script to analyse pneumatic GD volumes measured with a Pneumatron for *Olea europea*. In **(A)** the vulnerability curve and the traits estimated from the sigmoidal curve (in blue) are shown. The values in red are the measured values at 12, 50, and 88% PGD (percentage of gas discharged), while the values in blue represent the corresponding estimated values. The water potential corresponding to 50% PGD is shown as the measured (red triangle) and the estimated (blue circle) value. In **(B,C)** the absolute amount of gas discharged (raw data) is shown as a function of water potential **(B)** and time **(C)**. In **(D)** the estimated water potential for every 15 min is shown as a function of time.

The script will also save three figures: the vulnerability curve (Figure 6A), the volume of gas discharged versus water potential (Figure 6B), and the volume of gas discharged versus time (Figure 6C). By comparing the vulnerability curve (Figure 6A) with the gas discharge curves (Figures 6B,C), and the interpolation of the water potential over time (Figure 6D), it is possible to infer problems regarding leakages or water potential measurements. Also, a file with the summarised results will be saved (results.csv). The script estimates the Ψ_50_, Ψ_88_, and Ψ_12_ by fitting a sigmoidal curve (p50.pad, p12.pad, and p88.pad results, following Pammenter and Van der Willigen (1998), or from the nearest data point measured directly by the Pneumatron (p50_near, p12_near, and p88_near results).

### Constructing Vulnerability Curves Using an Excel Spreadsheet

Vulnerability curves can be constructed in a fast and easy way because no time-consuming measurements or analyses are required. The file “data_example_pneumatron.xlsx” can be used to analyse the pneumatic data (Supplementary Material 2). A complete tutorial to use this excel file is shown in Supplementary Material 1. After curve fitting, vulnerability traits can be estimated in Microsoft Office Excel^®^, as shown in Supplementary Material 2.

## Discussion

The construction manual, the operational details, and the software presented in this paper provide relevant and important information to users who want to apply the Pneumatron to estimate xylem embolism resistance. Vulnerability curves based on pneumatic measurements have been compared in various studies against various alternative methods (Pereira et al., 2016; Bittencourt et al., 2018; Zhang et al., 2018; Jansen et al., 2020; Guan et al., 2021; Paligi et al., 2021). These earlier experiments showed that the pneumatic method provides accurate estimations of embolism resistance, similar to the bench dehydration method, centrifuge-flow measurements (ChinaTron or cavitron; Cochard et al. (2005) and Wang et al. (2014)), and the optical method (Brodribb et al., 2016). A comparison of Ψ_50_ data based on the Pneumatic method with alternative approaches for 51 angiosperm species shows a strong and highly significant correlation (*R* = 0.71, *n* = 57 specimens; including data from Pereira et al., 2016, 2021; Zhang et al., 2018; Guan et al., 2020, 2021; Sergent et al., 2020; Chen et al., 2021; Paligi et al., 2021). Measuring errors and incorrect estimations of Ψ_50_ values can easily be identified when the recommendations outlined in this manuscript are considered. The removal of incorrect Ψ_50_ values in the above references considerably improves the correlation between P_50_ values based on the pneumatic method and other methods. Moreover, the Unit Pipe Pneumatic model indicates that 91% of the gas volume extracted in 15 s of simulation comes from the first two series of intact and embolised vessels, while only 9% of the gas extracted results from xylem sap (Yang et al., 2021), which is saturated or supersaturated with gas (Schenk et al., 2016). This modelling exercise also showed that the Pneumatron may underestimate embolism resistance by 2–17%, with a typical measuring error of 0.11 MPa for Ψ_50_ values. Such measuring accuracy is better or at least equal to the typical measuring disagreement by various hydraulic methods (Cochard et al., 2013; Jansen et al., 2015). Empirical evidence also confirms that the highest accuracy of Ψ_50_ estimations with a Pneumatron corresponds to a measuring time of 15 s (Paligi et al., 2021).

The high measuring accuracy of the Pneumatron is mainly due to the fast axial diffusion of gas across intervessel pit membranes (Kaack et al., 2019; Yang et al., 2021), while radial diffusion across cell walls and between the xylem and the bark is extremely slow (Sorz and Hietz, 2006; Wang et al., 2015). Even if intervessel pit membranes are hydrated, with a maximum water volume fraction of ca. 80% (Zhang et al., 2020), diffusion across 200–1,000 nm thick pit membranes will be fast and within seconds (Yang et al., 2021). Considering the low costs, easy operation, and fast analysis, the Pneumatron has considerable advantages over most conventional, both hydraulic and non-hydraulic methods (Table 1). Because cut-open conduits need to be embolised before pneumatic measurements start, there is no artefact associated with cutting xylem under negative pressure (Wheeler et al., 2013). The manual pneumatic approach, however, could result in a considerably large measuring error due to unprecise recording of fast gas diffusion during the first seconds of gas extraction. Because the accuracy of pneumatic measurements depends strongly on the amount of gas that diffuses from embolised conduits into the discharge tube during the first seconds, we recommend users to work with a Pneumatron device instead of the manual pneumatic approach. Moreover, less data points are collected with the semi-automated mode, and the connection and disconnection of samples to the apparatus may change the leakage rates and tubing volume, which affects the measurement precision and the correct detection of the minimum and maximum gas discharge (GD_min_ and GD_max_) plateaus (Figure 4).

**TABLE 1:** Overview of the most common methods used to estimate xylem embolism resistance with the technical advantages, disadvantages, and key references.

Method	Advantages	Disadvantages	References
Bench dehydration	Cheap	Time-consuming; plenty of plant material needed; cutting-artefact; wounding artefact; destructive	Sperry et al., 1988; Tyree et al., 1992; Bréda et al., 1993; Wheeler et al., 2013; De Baerdemaeker et al., 2019; Bonetti et al., 2020
Centrifuge and flow-centrifuge	Fast; measurements rely on water transport under negative pressure; centrifugal forces determine xylem water potential	Open-vessel artefact; expensive; self-construction is technically challenging; hydraulic artefacts; destructive; water potential gradient within a single sample	Alder et al., 1997; Cochard et al., 2005; Torres-Ruiz et al., 2014, 2017; Wang et al., 2014; Peng et al., 2019
Air-injection (double ended cavitation chamber)	Relatively fast	Effervescence; vessel length exceeds cavitation chamber length; hydraulic artefacts; destructive	Cochard et al., 1992; Gullo and Salleo, 1992; Yin and Cai, 2018
Acoustic emissions	Expensive sensors; non-destructive	Data analysis time-consuming and not straightforward	Milburn and Johnson, 1966; Tyree et al., 1984; Nolf et al., 2015
Optical vulnerability	Cheap; applicable to leaf xylem and the outermost xylem of roots and stems; non-destructive	Time-consuming image analysis; mainly two-dimensional view of embolism	Brodribb et al. (2016) http://www.opensourceov.org/
MicroCT	Noninvasive imaging; 3D imaging of the xylem anatomy at high resolution	Extremely expensive; limited beam-time availability; time-consuming image analysis; multiple samples might be needed to construct a vulnerability curve; scanning volume relatively small	Choat et al., 2016; Klepsch et al., 2018; Gauthey et al., 2020
Magnetic resonance imaging	Non-destructive imaging; quantification of flow	Low resolution; expensive	Hochberg et al., 2016; Bouda et al., 2019; Meixner et al., 2020
Pneumatron	Cheap and straightforward operation, fast data analysis accurate estimation of the gas volume in embolised conduits due to fast diffusion	May not be applicable to gymnosperms with a torus-margo; destructive; no visual, anatomical information	Pereira et al., 2016, 2020a; Bittencourt et al., 2018; Zhang et al., 2018; Paligi et al., 2021; Yang et al., 2021

*Except for the centrifuge and flow-centrifuge, xylem water potential measurements are not considered.*

Yet, users should understand the pneumatic principles, which are different from hydraulic measurements, and should be familiar with the basics that determine gas diffusion kinetics. These physical laws include the ideal gas law, Henry’s law for gas concentration partitioning between liquid and gas phases at equilibrium, and Fick’s law for diffusion. When the Pneumatic method is incorrectly applied, data are likely misinterpreted (Sergent et al., 2020; Chen et al., 2021; Pereira et al., 2021). The two most common mistakes include incorrect adjustment of the discharge tube volume, and the lack of stable gas discharge measurements at the beginning and the end of pneumatic experiments (GD_min_ and GD_max_). Since pneumatic vulnerability curves are normalised against GD_min_ and GD_max_, it is crucial to have reliable values, with a stable minimum and maxium plateau (Figure 5). The consequence of not adjusting or incorrectly adjusting the volume of the discharge tube is a relatively high measuring error. When the discharge tube is too large or too small, the difference between the minimum and maximum air discharge volumes will be too small, resulting in relatively high measurement uncertainty (Jansen et al., 2020).

The main advantages of the Pneumatron device for constructing vulnerability curves are its low construction costs (<€100), the easy, automated operation, the fast analysis of pneumatic data, and its measuring accuracy. These issues are especially relevant when measurements on a large number of samples or species are desired. The device can also be used under remote field conditions, although water potential measurements may then become more challenging than pneumatic measurements. Moreover, vessel dimensions do not affect the precision of embolism resistance based on pneumatic measurements, as long as the discharge tube volume has been adjusted properly. The end-walls are the main resistance for gas diffusion between embolised vessels as the lumen resistance is negligible. Thus, gas can be extracted from three or four vessels in series, and this extraction is independent of the vessel length (Yang et al., 2021). This means that the Pneumatron can also be applied to liana’s and species with long vessels. In fact, gas diffusion becomes more precise for wide vessels as predicted by the Unit Pipe Pneumatic model (Yang et al., 2021). Ring-porous species, however, may need to be tested carefully because earlywood vessels in growth rings that are more than 1 year old are not only dysfunctional after 1 year, but also long and wide, and can be plugged with tyloses (Sano et al., 2011). Tyloses are likely to slow down gas diffusion rates. One solution to work with ring-porous species could be to glue off the xylem of previous growth rings (Zhang et al., 2018). Species that secrete resin, mucilage, oil or other substances, could also be problematic because their secretion at the cut end may prevent gas extraction. Removing a thin slice at the cut-open xylem every 2 or 3 h before a new measurement is taken may be a solution, but needs further experimental testing. It should also be tested if the Pneumatron can be applied to herbaceous plants and woody monocots.

A disadvantage is that the method is destructive and requires cutting open xylem tissue. Unlike hydraulic measurements, however, air entry of cut-open conduits should be aimed for as cut-open conduits function as an extension of the discharge tube. In case xylem samples are under positive xylem pressure (Schenk et al., 2020), xylem sap will come out at the cut-open end, which will not only be problematic for pneumatic measurements, but also for hydraulic methods. Other forms of wounding response are likely slow and unlikely to affect the vessel dimensions during dehydration. Special attention should also be paid to non-xylem tissue such as pith, which may undergo shrinkage during dehydration, or cracks could be formed. Cracks or shrinkage, however, are typically not problematic when samples with relatively small pith proportions are used. Even if air from the pith tissue is extracted, there is only a problem when the gas volume extracted from the pith increases considerably over time, which is unlikely due to slow diffusion through this tissue. Also, cracks will only affect gas extraction amounts if these are directly connected to cut-open xylem conduits or the cut xylem area.

So far, an automated Pneumatron device has not been applied yet to gymnosperms that possess a torus-margo pit membrane. The manual pneumatic approach suggested that torus-bearing species can be problematic (Zhang et al., 2018), probably due to fast pit membrane aspiration (Zelinka et al., 2015; Schulte and Hacke, 2020), which may prevent gas extraction from intact, embolised tracheids. The manual pneumatic method, however, has been successfully applied to vesselless angiosperms (Pereira et al., 2016), which have wood composed of tracheids only, but no torus-margo pit membranes. It is possible that modification of the applied vacuum, discharge tube volume, and/or extraction time could make the automated Pneumatron applicable to gymnosperm xylem, and this needs to be tested.

In addition to studies on xylem embolism, the Pneumatron has also been used to estimate vessel length distribution in an easy and much faster way (Pereira et al., 2020b) than conventional methods (Williamson and Milburn, 2017; Link et al., 2018). This is possible by easy modification of the reservoir volume and by running the Pneumatron in a semi-automated way. The data analysis is also straightforward by using an R-script, and thus, makes it feasible to obtain fast measurements of vessel length distributions, and to investigate the non-random distribution of vessel ends near nodes, side branches, stem-petioles transitions, etc. Pneumatic estimations of the hydraulically weighted vessel length have been validated against the silicon-injection method for five species, and the air-injection method of Cohen et al. (2003) for seven species (Yang et al., 2021).

We hope this manual makes the Pneumatron accessible to many plant biologists as a high quality, low-cost, versatile tool to study embolism resistance in angiosperms, and gas diffusion kinetics in general. We emphasize that the protocol presented here includes recommendations only, while users can easily modify the device and software programmes, depending on the experimental requirements. It is, for instance, possible to modify and improve the hardware and software for various types of gas exchange measurements, or to integrate additional sensors into the system. Users who prefer not to construct their own device are reminded that a user-friendly Pneumatron is commercially available (Plantem–Plant and Environment Technologies, Campinas, Brazil). Different versions have been used satisfactorily so far, using other components than proposed here (Pereira et al., 2020a; Wu et al., 2020), or increasing the number of samples that are simultaneously measured (Pereira et al., 2020a). Any modification of the hardware, such as the changes needed to allow simultaneous measurements on multiple samples, will require further adaptation to the software as well as the R-script for data analysis. We encourage users to share modified and updated versions with each other in a collaborative spirit as this would further improve pneumatic measurements on plant tissues, and promote insights about gas exchange of plants.

## Data Availability Statement

The programmes for the Pneumatron can be downloaded from https://github.com/Pneumatron/software.

## Author Contributions

CT, LP, MM, and XG wrote the first version of the manuscript. LP developed the R-script and the Arduino software, this latter with contributions from CT and PB. CT created the calibration protocol, calibration software, and the troubleshooting section. XG and RR wrote the protocol and the template for the Excel analysis. The manuscript received substantial contributions from SJ, RR, PB, and RO. All authors contributed to the article and approved the submitted version.

## Conflict of Interest

The authors declare that the research was conducted in the absence of any commercial or financial relationships that could be construed as a potential conflict of interest.

## Footnotes

1 https://www.arduino.cc/en/Main/Software

2 https://github.com/Pneumatron/software

3 https://github.com/Pneumatron/analysis

## Supplementary Material

Supplementary Figure 1 Schematic connection of the electronic components in a Pneumatron device.

Supplementary Table 1 Component list; Tutorial to use the excel file for data analysis (Supplementary Figures 2–10).

Supplementary Material 2 Excel file for data analysis.
