# Supplementary material for: A User Manual to Measure Gas Diffusion Kinetics in Plants: Pneumatron Construction, Operation, and Data Analysis
Source: Front Plant Sci. 2021 Jun 7;12:633595. doi: 10.3389/fpls.2021.633595 (PMC8216216; doi:10.3389/fpls.2021.633595)
Supplement: Supplementary Figure 1 — Schematic connection of the electronic components in a Pneumatron device. [file Data_Sheet_1.zip › Supplementary Material 1.PDF]

frontiers

Supplementary Material

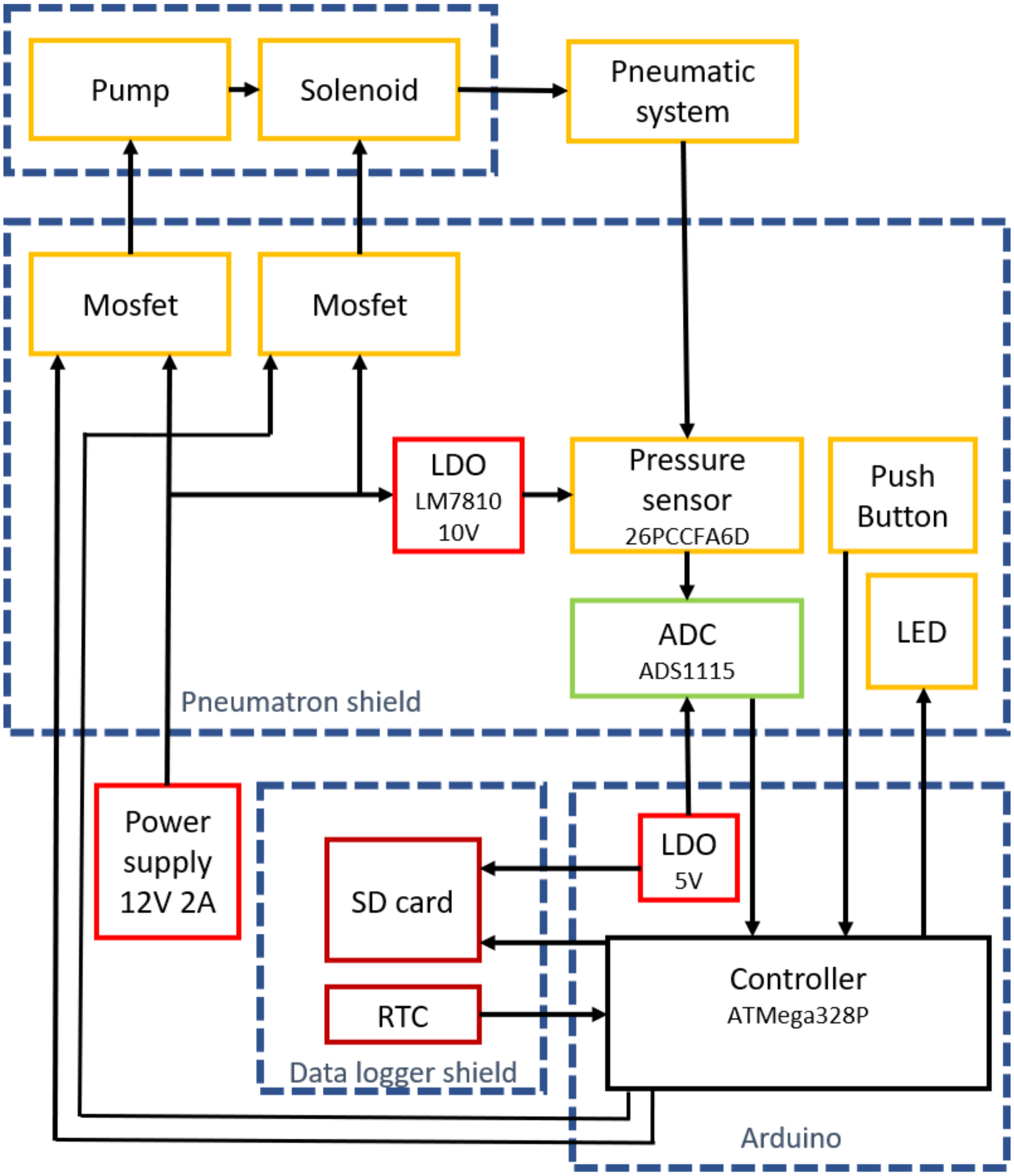

Figure S1 – Schematic connection of the electronic components in a Pneumatron device.

Table S1 – Component list

	**Component**	**Manufacturer**	**Catalog/part code**	**Key specifications**
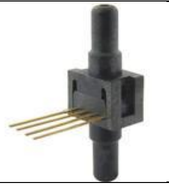	Differential pressure sensor	Honeywell	26PCCFA6D	15 Psi<br><br>100 mV spam
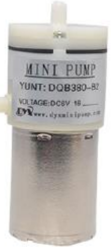	Mini vacuum pump	Dyx, Shenzhen, China	DQB380-FB2	12 V, 700mA maximum
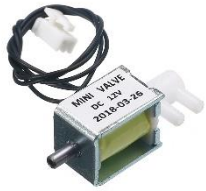	Three-way solenoid valve<br><br>(12 volts)	Dongguan City-Electric Co., Dongguan, China	Fa0520F	12 V, 500 mA maximum
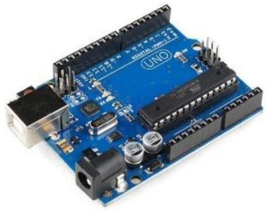	Arduino UNO			
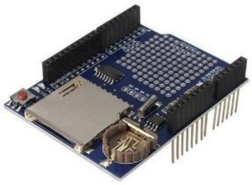	Data logger shield			DS1307<br><br>MicroSD adapter plus level shifters
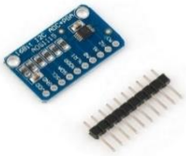	Analogic to digital converter 16 bits		ADS1115 breakout board	Configured to address 0x48

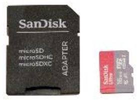	Micro-SD memory card and SD adapter (see data logger module)			SD or SDHC
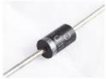	2 Schotksy Diode		1N4007	Used as flyback diode; fast response, low forward voltage.
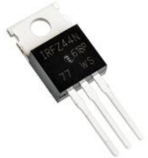	2 N-ChannelMOSFET		IRFZ44NPBF	5 V logic level; Igs >= 3A
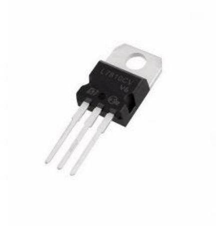	Voltage regulator		L7810	
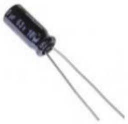	Eletrolytic Capacitor 10UF 63V			
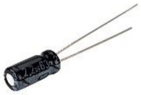	Eletrolytic Capacitor 2.2UF 63V			
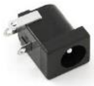	DC Power Supply Jack Socket Female Panel Mount Connector			
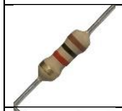	Carbon resistor 1K			
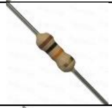	Carbon resistor 10K			
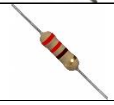	Carbon resistor 220 ohms			
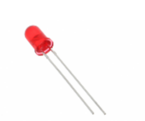	Light Emitting Diode			

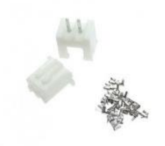	Pin header, housing and cable crimp		JST-XH	Vertical, 2.5 mm pitch (if XH)
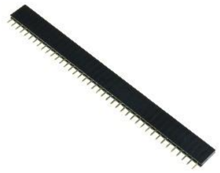	Single Row Straight Female Pin Receptacle			2.5 mm pitch
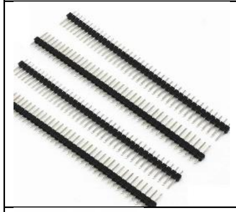	Single Row Straight Male Pin Header Strip			
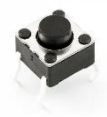	Push button			6 x 6 mm, normal open
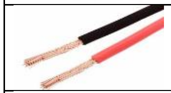	Wires (red and black)	20 cm each		AWG <=28
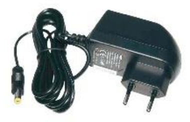	Power source			12 VDC, 2 A
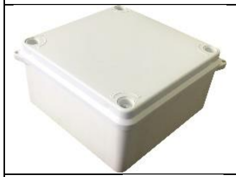	Plastic enclosure			10x10x5 cm
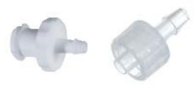	Adapter Luers	Cole-Parmer	EW-30800-06 and EW-30800-24	
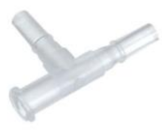	Luer Adapter Tees, Male x Male x Female	Cole-Parmer	GY-45508-75	
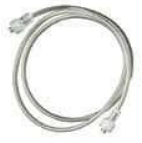	Rigid Tubing	Cole-Parmer	EW-30600-62	

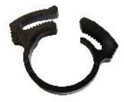	Plastic clamps	Cole-Parmer	RZ-06832-02	
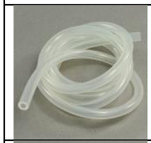	Silicone tubing (2 mm Internal Diameter)			
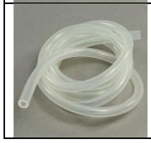	Silicone tubing (4 mm ID and 6 mm OD)			

Troubleshooting

Some of the following issues could be problematic when constructing or operating Pneumatron devices. The most common problems are related to leakage between components, malfunctioning of the data logger shield, or the solenoid valve. On the other hand, problems related to the Arduino board, electric components (such as Mosfets, resistors, etc.), or the vacuum pump are rare.

A. Nothing works

- Check the power supply.
- Check connections on the different shields and boards.
- Check that the pin connections are matching their counterparts.
- Check the pressure sensor and/or make sure the ADC 16bits converter is accurately connected.
- Connect the Pneumatron to a computer, open the Arduino software and then open the serial monitor. Read the potential error messages if nothing appears to work (See section D).

B. Frequent or continuous pumping of the vacuum pump: leakage

- If the main source of leakage has been identified to be the solenoid valve, consider replacing it.
- An alternative, but uncommon source of leakage can be the T junction.
- A leakage may also come from a not accurately inserted tube. Check if the solenoid valve and the pump are properly connected (following the correct orientation).
- The pump screws may be loose, and thus the pump cannot create the pressure requested by the programme.

C. No pumping at all when powered

- Check if the pressure is near zero and not between 10 and 50 kPa. The solenoid valve may not open properly at the end of a measurement and the pressure does not return to atmospheric pressure. Consider increasing the tubing volume between the pump and solenoid valve or replace the solenoid.
- Check the connection to the pump.
- Check if the pressure sensor and/or the ADC 16bits converter are correctly connected.
- Check if the programme has been properly uploaded on the board.

D. No data recorded or unreasonable values (i.e. all values are identical or outliers)

- The SD card may be set on locked, corrupted, unformatted, or not fully inserted in the data logger shield. Unlock the card, format the card, or check if the card is fully inserted and in the right orientation.
- Check the pressure sensor and/or make sure the ADC 16bits converter is correctly connected.
- There could be a problem with the data logger shield. The pins may be not well connected, or another problem that is hard to detect may occur. Consider replacing it.
- An error in the time recording can be due to a different RTC (Real Time Clock). The programme works with the RTC DS1307, and the data logger should be checked. If the

inscription PCF8523 is shown, change RTC_DS1307 to RTC_PCF8523, and change rtc.isrunning by rtc.initialized.

E. The pump seems to run too slowly

- Check the power supply. The voltage or power might be too low. Consider replacing the power supply.

F. The Arduino flashes rapidly but nothing else works.

- This problem occurs when the voltage from the power supply is inferior to the optimal value. Consider changing the power supply.
- Alternatively, too many Mosfet transistors and/or too many pressure sensors are connected. Consider supplying the voltage to the Arduino from another source.

Tutorial to use the excel file for data analysis.

1.1 Get the data.

1.1 Import the csv file into Excel software.

1.2 Re-arrange the data: split the single column to separate ones.

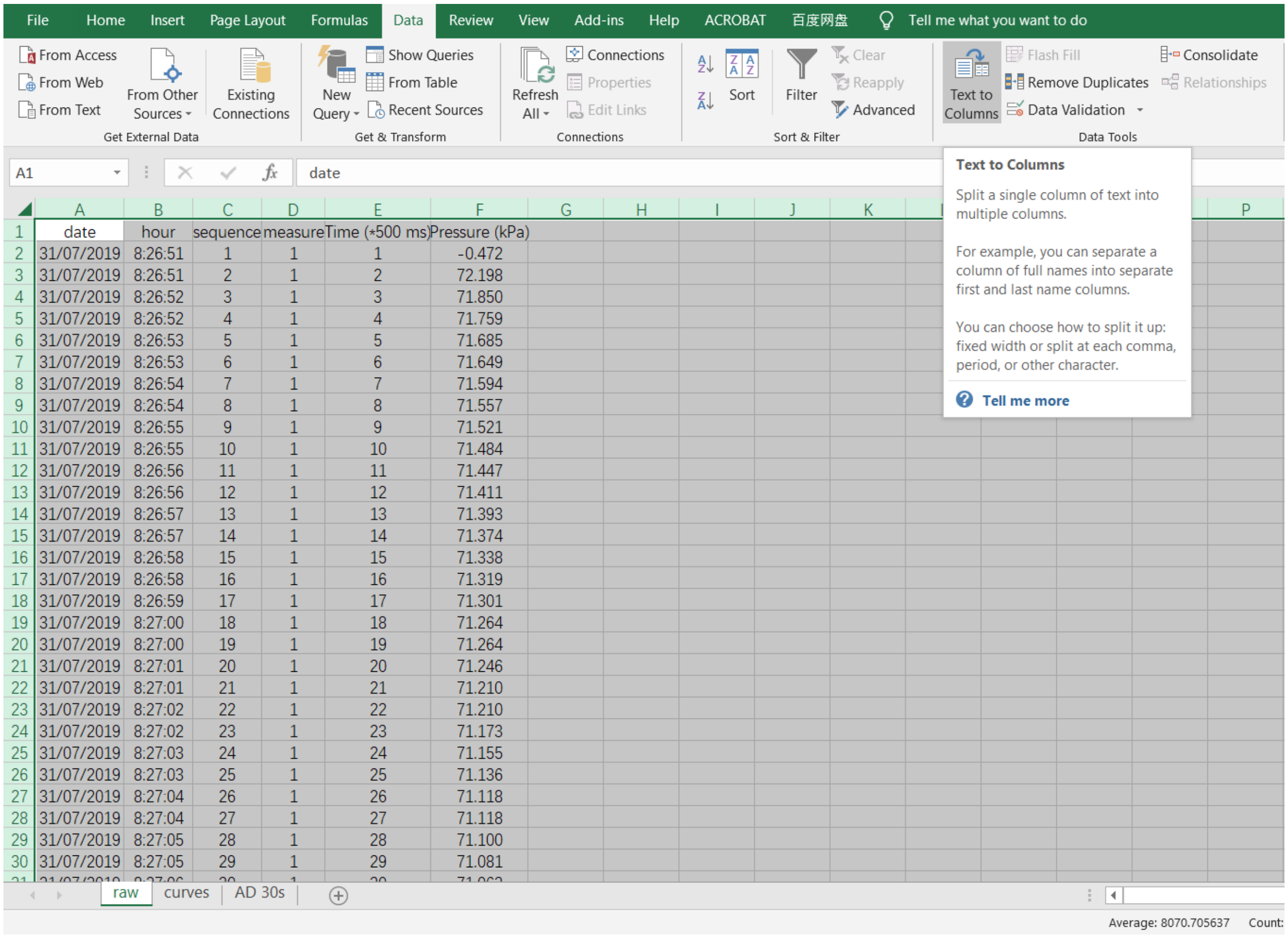

Supplementary Figure S2: Re-arranging data

1.3 Add titles to column D, E and F with “Measure”, “Time (*500 ms)” and “Pressure (kPa)”

“Measure” gives the information of the exact round of measurement that is taking. “Time” tells the sequence of records during one measurement, and “Pressure” tells the corresponding pressure, which is recorded at every 500 ms.

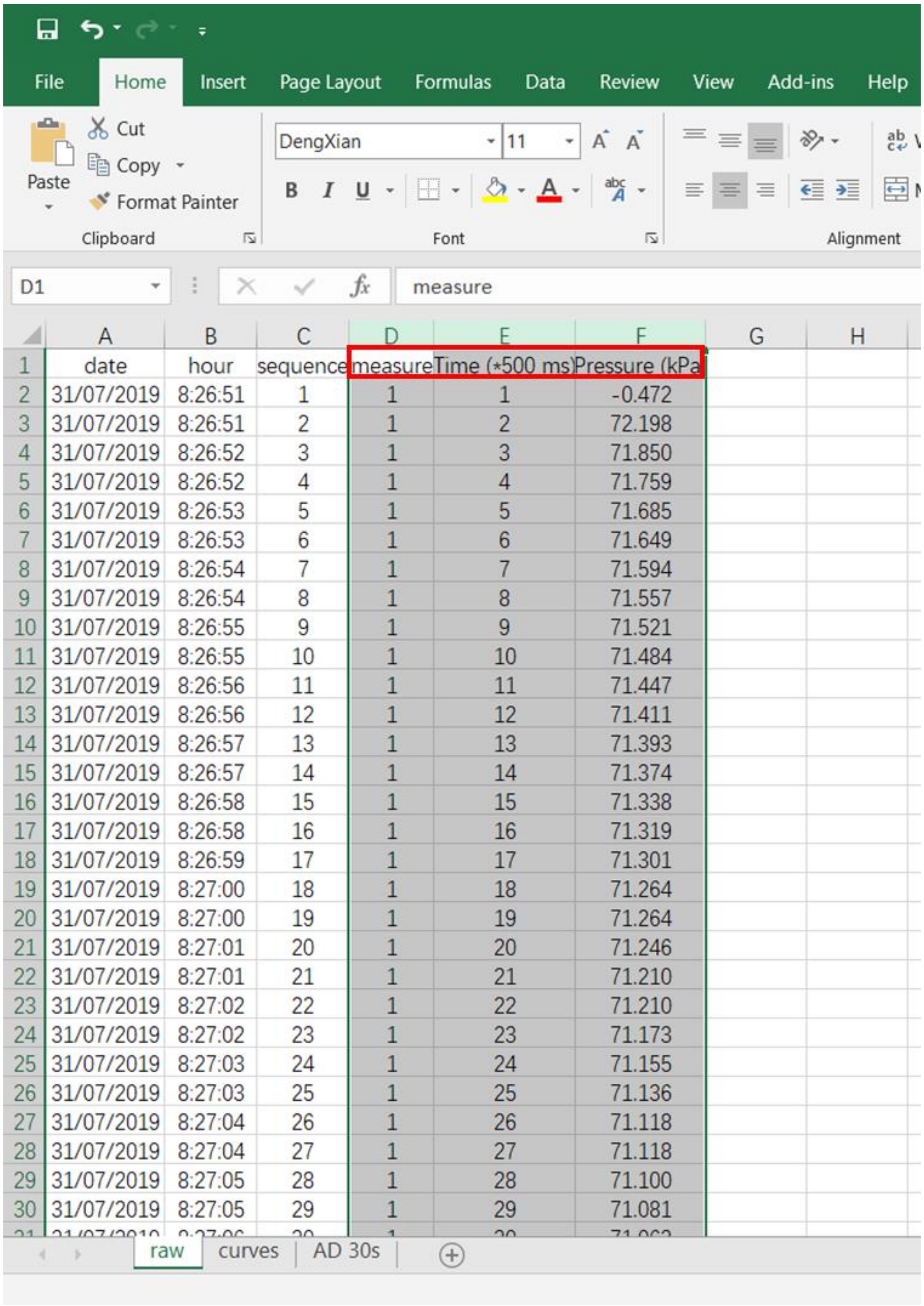

Supplementary Figure S3: Adding headers

1.4 Select the three columns named "Measure", "Duration", "Pressure". Go to "Insert" and choose "PivotTable".

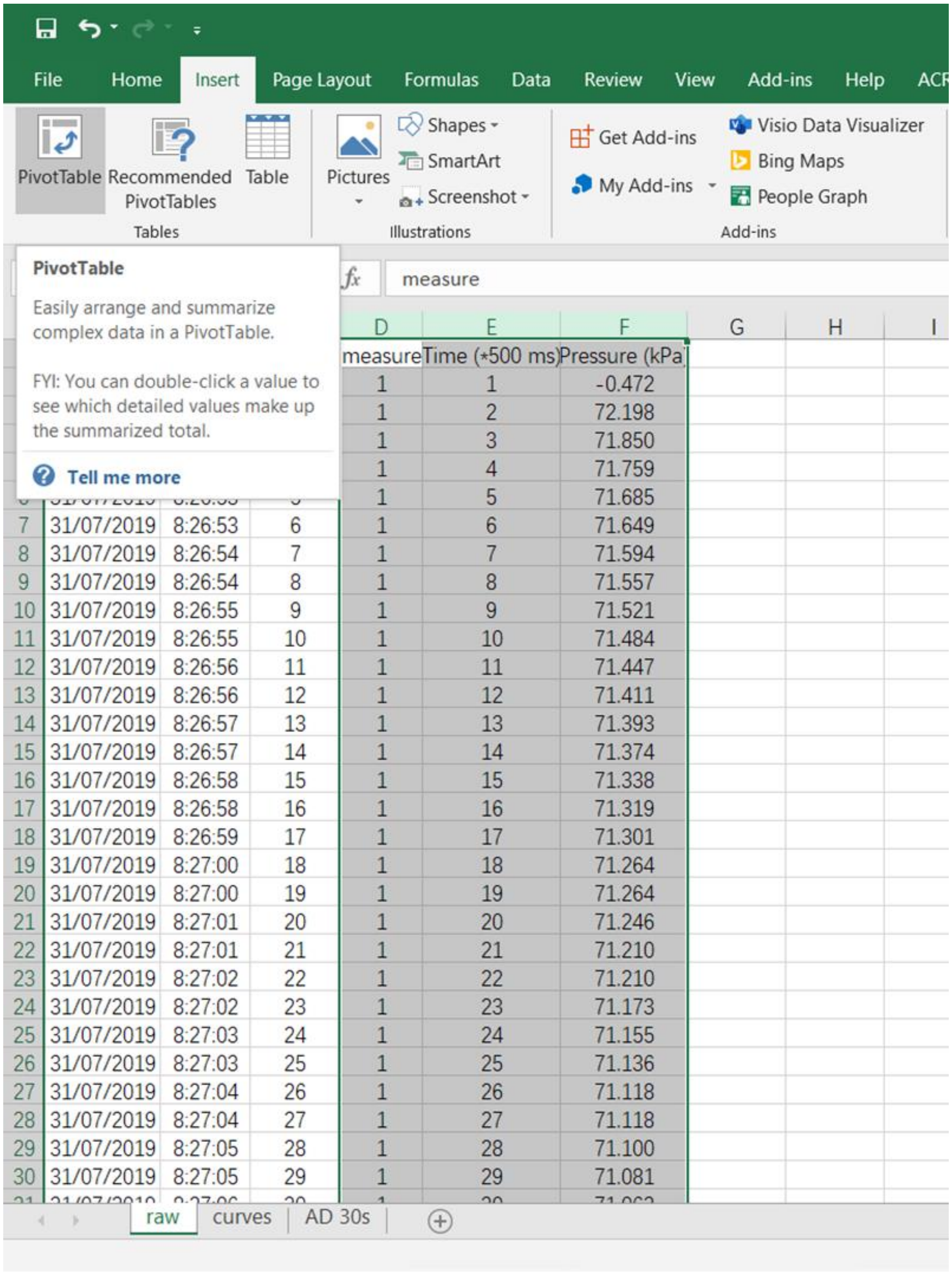

Supplementary Figure S4: Re-arranging columns

1.5 Create a new sheet and drag columns.

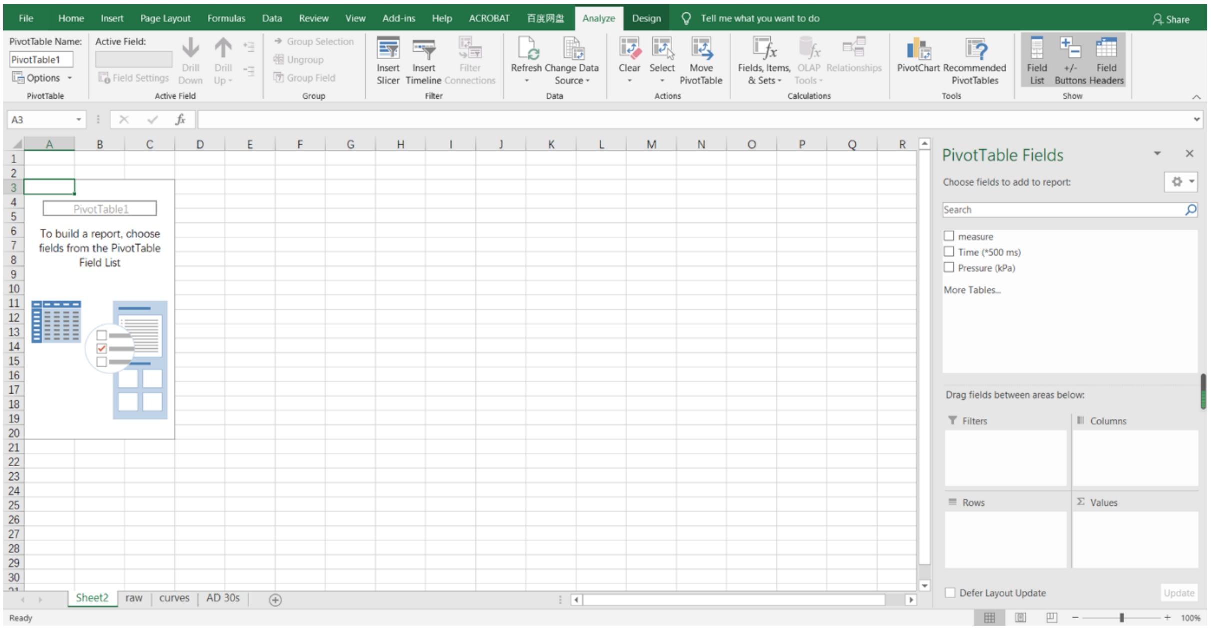

Supplementary Figure S5: New sheet

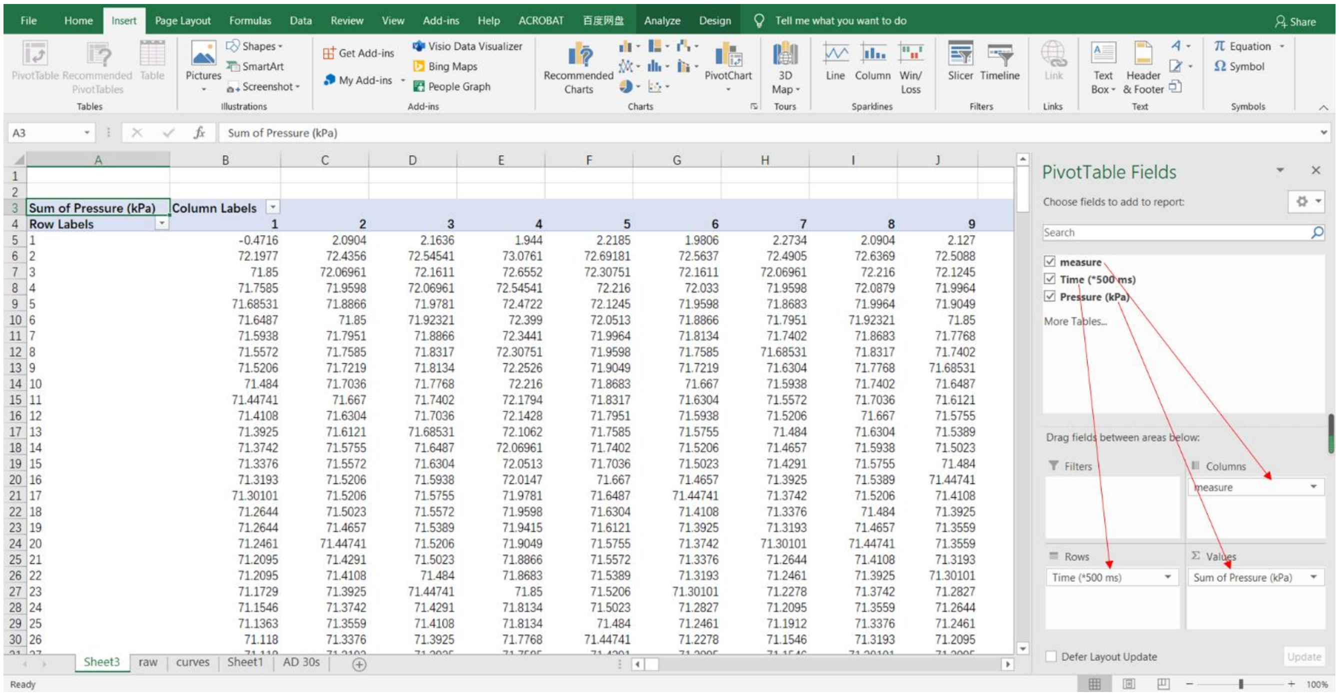

Supplementary Figure S6: Dragging columns

Here, you can see the "PivotTable Fields" on the right side of Figs S5 and S6. Drag "Measure" to Column, "Time" to Row and "Pressure" to Value.

1.6 Select the whole row data at Row 6 and Row 63.

Select all data from Row 7 and Row 64, and copy the data to a new sheet. These are the data measured 1.5 s and 30 s after pumping, and they are taken as the initial pressure (P_i) and final pressure (P_f).

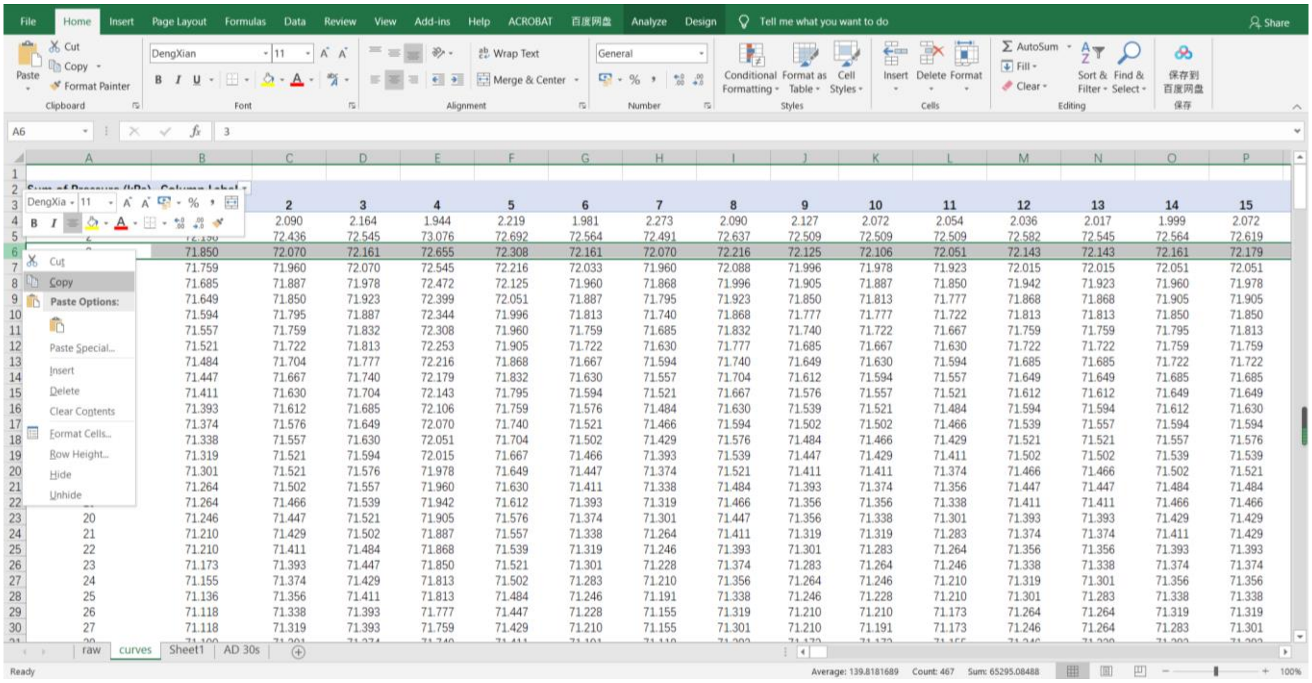

Supplementary Figure S7: Selecting rows

1.7 For the next steps, you may use the data_example.xlsx file in Supplementary Material 7.

1.8 Calculate the volume of gas discharged (GD) from a branch as well as the percentage of air discharged (PGD).

Ideal gas calculations: The ideal gas law is applied to calculate the volume of gas discharged. The moles of gas (Δn) discharged are first calculated as follows:

$$\Delta n = \frac{P_i V_r}{R T} - \frac{P_f V_r}{R T} \tag{1}$$

where V_r is reservoir volume (mL), which corresponds to the volume of the tubing from the pressure sensor to the tube connection of the sample; T is the absolute temperature of gas in Kelvin, and R is the ideal gas constant (8.3144621 J mol^{-1} K^{-1}). Convert the moles of gas discharged to volume, find the largest and smallest volume of air discharged, and calculate PGD according to equation 2:

$$PGD = 100 \left(\frac{GD - GD_{min}}{GD_{max} - GD_{min}}\right) \tag{2}$$

J2 =(((I2*1000)*(E2*10^-6))/(8.3144621*293.15))-(((H2*1000)*(E2*10^-6))/(8.3144621*293.15))

	E	F	G	H	I	J	K	L	M
1	Reservoir volume (mL)	Pi (kPa)	Pf (kPa)	Absolute Pi (kPa)	Absolute Pf(kPa)	GD (mol)	GD (uL)	PGD (%)	Xylem water potential (MPa)
2	1.305	72.161	70.953	27.839	29.047	6.467E-07	16.083	0.0	-0.15
3	1.305	72.161	70.953	27.839	29.047	6.467E-07	16.083	0.0	-0.21
4	1.305	72.161	70.953	27.839	29.047	6.467E-07	16.083	0.0	-0.27
5	1.305	72.655	71.264	27.345	28.736	7.446E-07	18.520	0.9	-0.33
6	1.305	72.308	70.935	27.692	29.065	7.349E-07	18.277	0.8	-0.38
7	1.305	72.161	70.697	27.839	29.303	7.838E-07	19.495	1.3	-0.44
8	1.305	72.070	70.642	27.930	29.358	7.642E-07	19.008	1.1	-0.50

K2 =(J2*8.3144621*293.15/98000)*1000*1000*1000

	E	F	G	H	I	J	K	L	M
1	Reservoir volume (mL)	Pi (kPa)	Pf (kPa)	Absolute Pi (kPa)	Absolute Pf(kPa)	GD (mol)	GD (uL)	PGD (%)	Xylem water potential (MPa)
2	1.305	72.161	70.953	27.839	29.047	6.467E-07	16.083	0.0	-0.15
3	1.305	72.161	70.953	27.839	29.047	6.467E-07	16.083	0.0	-0.21
4	1.305	72.161	70.953	27.839	29.047	6.467E-07	16.083	0.0	-0.27
5	1.305	72.655	71.264	27.345	28.736	7.446E-07	18.520	0.9	-0.33
6	1.305	72.308	70.935	27.692	29.065	7.349E-07	18.277	0.8	-0.38
7	1.305	72.161	70.697	27.839	29.303	7.838E-07	19.495	1.3	-0.44
8	1.305	72.070	70.642	27.930	29.358	7.642E-07	19.008	1.1	-0.50

L2 =(100*(K2-(MIN(K:K))))/((MAX(K:K))-(MIN(K:K)))

	E	F	G	H	I	J	K	L	M
1	Reservoir volume (mL)	Pi (kPa)	Pf (kPa)	Absolute Pi (kPa)	Absolute Pf(kPa)	GD (mol)	GD (uL)	PGD (%)	Xylem water potential (MPa)
2	1.305	72.161	70.953	27.839	29.047	6.467E-07	16.083	0.0	-0.15
3	1.305	72.161	70.953	27.839	29.047	6.467E-07	16.083	0.0	-0.21
4	1.305	72.161	70.953	27.839	29.047	6.467E-07	16.083	0.0	-0.27
5	1.305	72.655	71.264	27.345	28.736	7.446E-07	18.520	0.9	-0.33
6	1.305	72.308	70.935	27.692	29.065	7.349E-07	18.277	0.8	-0.38
7	1.305	72.161	70.697	27.839	29.303	7.838E-07	19.495	1.3	-0.44
8	1.305	72.070	70.642	27.930	29.358	7.642E-07	19.008	1.1	-0.50

Supplementary Figure S8: Volume calculations

1.9 Plot PGD against time

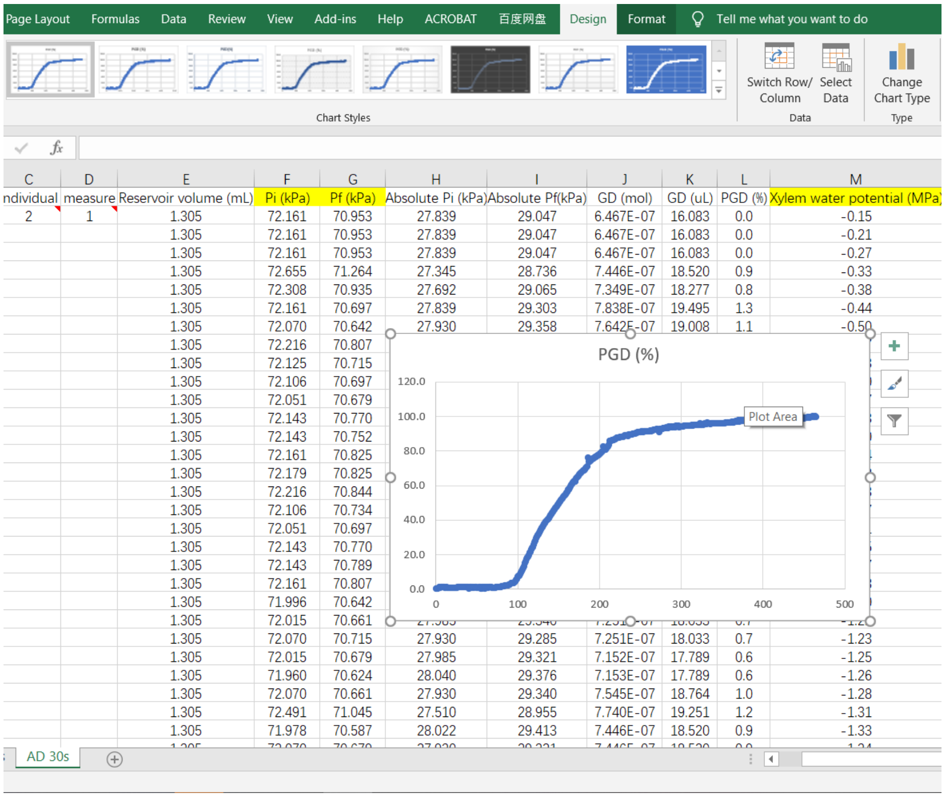

Supplementary Figure S9: Plotting PGD against time

1.10 Include xylem water potential measurements

Take the xylem water potential measurements (e.g. pressure chamber or psychrometer measurements). Plot xylem water potential data as X-axis and the corresponding PGD results as Y-axis to get a vulnerability curve. The pale blue line indicates a sigmoidal fitting, which can be applied by following the instructions given the template.

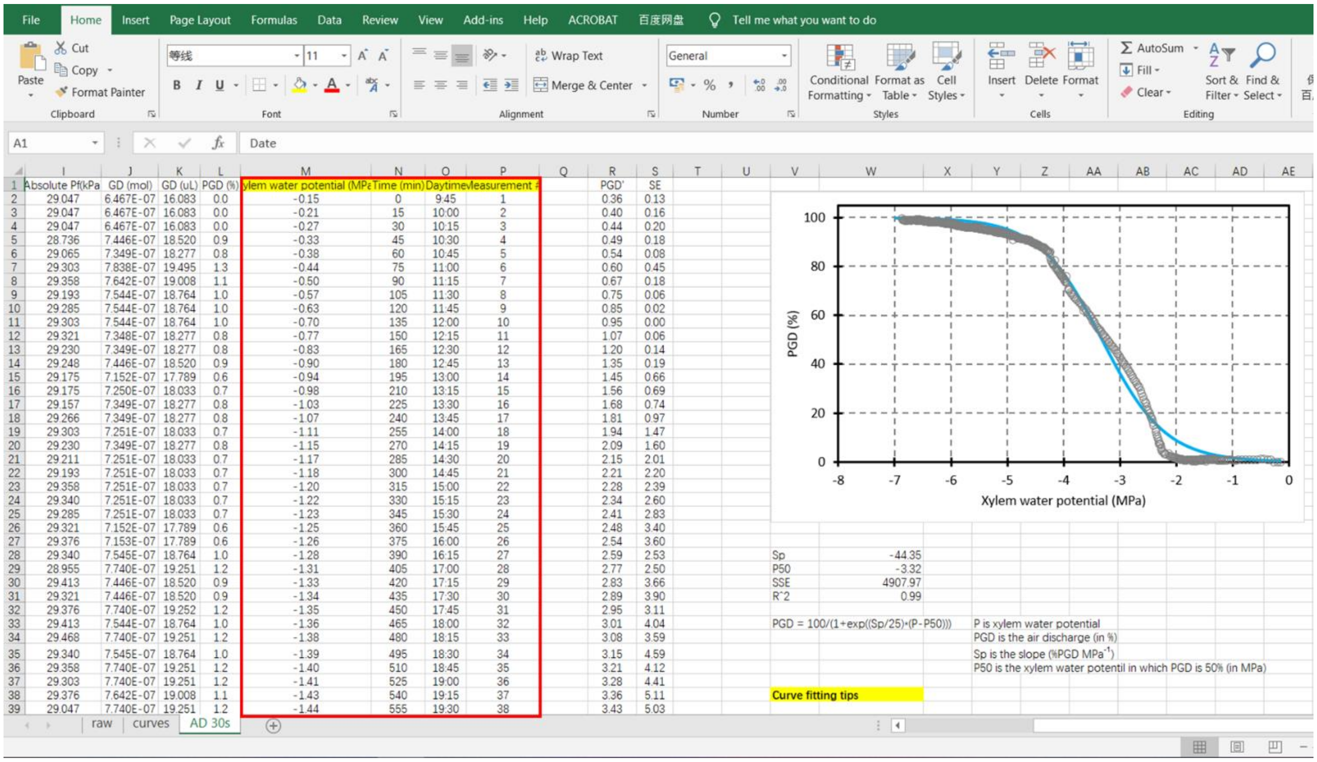

Supplementary Figure S10: Plotting PGD against corresponding xylem water potential data
